# Papillary Fibroelastoma of the Ascending Aorta

**DOI:** 10.1177/2324709619840377

**Published:** 2019-04-22

**Authors:** Karim M. Al-Azizi, Mohanad Hamandi, Ronald Baxter, Anita Krueger, Alexander W. Crawford, Michael William, Christopher Good, Nicolas Mead

**Affiliations:** 1 Baylor Scott & White, the Heart Hospital, Plano, TX, USA; 2 Geisinger Medical Center, Danville, PA, USA; 3 Geisinger Commonwealth School of Medicine, Scranton, PA, USA

**Keywords:** papillary fibroelastomas, ascending aorta

## Abstract

Papillary fibroelastomas are rare benign primary cardiac tumors. They are typically found on valvular surfaces, most commonly, the aortic valve. In this article, we report a case of papillary fibroelastoma arising from the sinotubular junction of the ascending aorta, a rare and unusual site causing an embolic stroke.

## Introduction

Papillary fibroelastomas (PFEs) are rare benign primary cardiac tumors, and clinical presentations can be varied. However, embolic events leading to neurological sequelae, such as transient ischemic attacks or strokes, are the most common presentations.^1^ Advancements in cardiac imaging have led to increasing diagnoses of PFEs. They are typically found on valvular surfaces with the aortic valve being the most common location.^1^ PFEs have also been described arising from unusual locations.^2^ Reported is a case of a probable PFE arising from the sinotubular junction of the ascending aorta, a rare and unusual site.

## Case Report

A 75-year-old white woman with history of hypertension and endometrial cancer was admitted for radical hysterectomy and hernia repair. Her intraoperative course was uneventful, but on postoperative day 2, she developed acute onset of right-sided weakness and was subsequently diagnosed with a stroke. There was no intracranial bleed on non-contrasted computed tomography imaging, but due to her recent surgery, she did not qualify for thrombolytic therapy and further neurologic workup was initiated. Magnetic resonance imaging of the brain showed multiple areas of restricted diffusion in the occipital and temporal lobes suggestive of acute embolic events.

**Figure 1.**
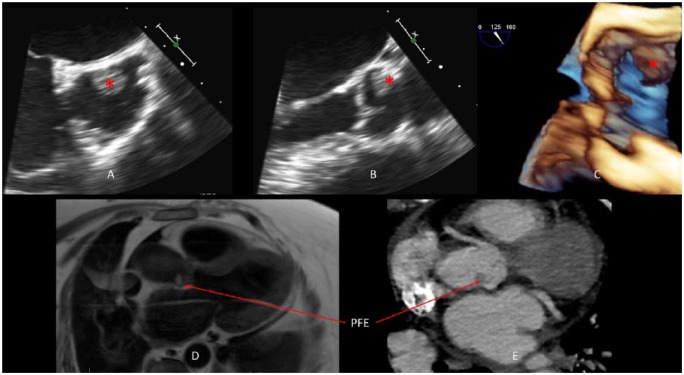
(A) Transesophageal echocardiogram (TEE) short-axis view of the aortic valve shows round echo-density mass (*), between the left and noncoronary cusps, in proximity to the left main ostium. (B) TEE long-axis view of the aortic valve as well as the ascending aorta demonstrates papillary fibroelastoma (PFE) location. (C) PFE noted on 3-dimensional TEE long-axis view of the aortic valve, aortic root, and ascending aorta. (D) T2-weighted cardiac magnetic resonance imaging confirms the density noted in the aortic root, attached to the sinotubular junction. (E) Computed tomography image of a prior scan suggestive of a mass in the aortic root.

During diagnostic workup to determine the embolic source, a transesophageal echocardiogram revealed no intracardiac thrombus but a 1.2 cm × 0.9 cm echo-density arising from the sinotubular junction of the ascending aorta was discovered. This lesion was in proximity to the left main coronary artery (Figure 1). Long- and short-axis views showed punctate calcifications within the stalk of the lesion and displayed an “anemone”-like appearance. Electrocardiographically, she was found to be in sinus rhythm with no evidence of arrhythmias.

Subsequently, a cardiac magnetic resonance imaging was performed for better tissue characterization. T2-weighted images confirmed the presence of a mass at the sinotubular junction, and a prior non-gated computed tomography scan of the chest showed a similar finding. A multidisciplinary team discussion with the patient and her family was held, and it was determined that she was at high risk for curative surgical resection due to multiple comorbidities. Therefore, pathological evaluation of this lesion was not possible. However, given the various imaging modalities used with concordant findings, a general consensus was reached that this lesion was most likely a PFE arising from the sinotubular junction. The patient was subsequently discharged to inpatient rehabilitation on anticoagulation and was unfortunately lost to follow-up.

## Discussion

Papillary fibroelastomas are the most common primary tumors of the cardiac valves.^3^ It is believed that the incidence of PFEs was previously underestimated until recent advancements in imaging modalities.^4^ In a retrospective cohort study by Gowda et al, usual locations of PFEs were the aortic valve (36%), the mitral valve (29%), the tricuspid valve (11%), and the pulmonary valve (7%).^1^ The involvement of extravalvular sites is especially rare.

There have been limited reports of unusual sites of PFEs in the past, specifically the ascending aorta.^2,5^ Advancements in multi-imaging modalities is likely the cause of the increasing incidence of PFEs. In patients with PFEs who present with embolic strokes and who are of acceptable operative risk, excision of the lesions at highly experienced surgical centers has been the standard of care.^4,6^ Furthermore, excision of PFEs through minimally invasive approaches have also been described.^7^

In this reported case, an embolic stroke occurred likely secondary to the PFE. Due to this association and her clinical presentation, surgical resection was indicated. Unfortunately, due to her comorbidities, she was deemed high risk for surgery.

It can be challenging to manage patients diagnosed with this cardiovascular tumor. Currently, depending on symptoms, size, and tumor mobility, either surgical resection or anticoagulation regimen is usually offered to those patients.^1,8^ More data are needed to help guide the nonsurgical management of high surgical risk patients with these lesions.
